# Supplementary material for: Differential retinal ganglion cell resilience to optic nerve injury across vertebrate species
Source: Front Neurosci. 2025 May 20;19:1596464. doi: 10.3389/fnins.2025.1596464 (PMC12129905; doi:10.3389/fnins.2025.1596464)
Supplement: Supplementary file 4 [file Data_Sheet_1.pdf]

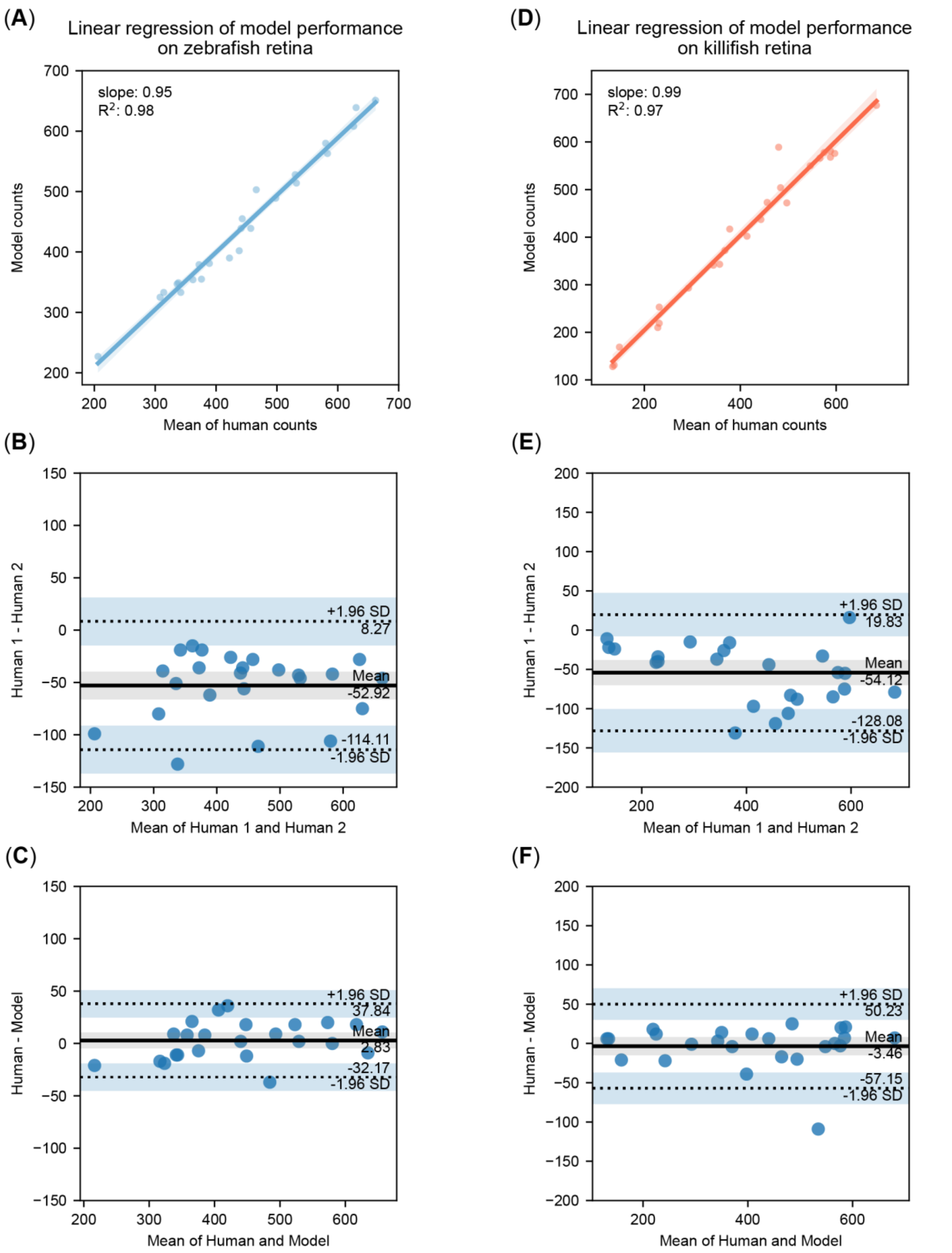

Supplementary figure 1: Performance metrics of zebrafish and killifish RGC counting models

(**A**) Linear regression of automated counts versus the average human count for zebrafish retinal ganglion cells (RGCs). The trained model shows very good performance.

(**B**) Bland-Altman plot comparing the counting bias of the two human counters for zebrafish RGCs. The intrinsic difficulty in counting dense RGCs leads to bias across multiple evaluators.

(**C**) Bland-Altman plot comparing the automated count versus the average human count for zebrafish RGCs. The model shows no bias compared to the average human count.

(**D**) Linear regression of automated counts versus the average human count for killifish RGCs. The trained model shows excellent performance.

(**E**) Bland-Altman plot comparing the counting bias of the two human counters for killifish RGCs. The intrinsic difficulty in counting dense RGCs leads to bias across multiple evaluators.

(**F**) Bland-Altman plot comparing the automated count versus the average human count for killifish RGCs. The model shows no bias compared to the average human count.

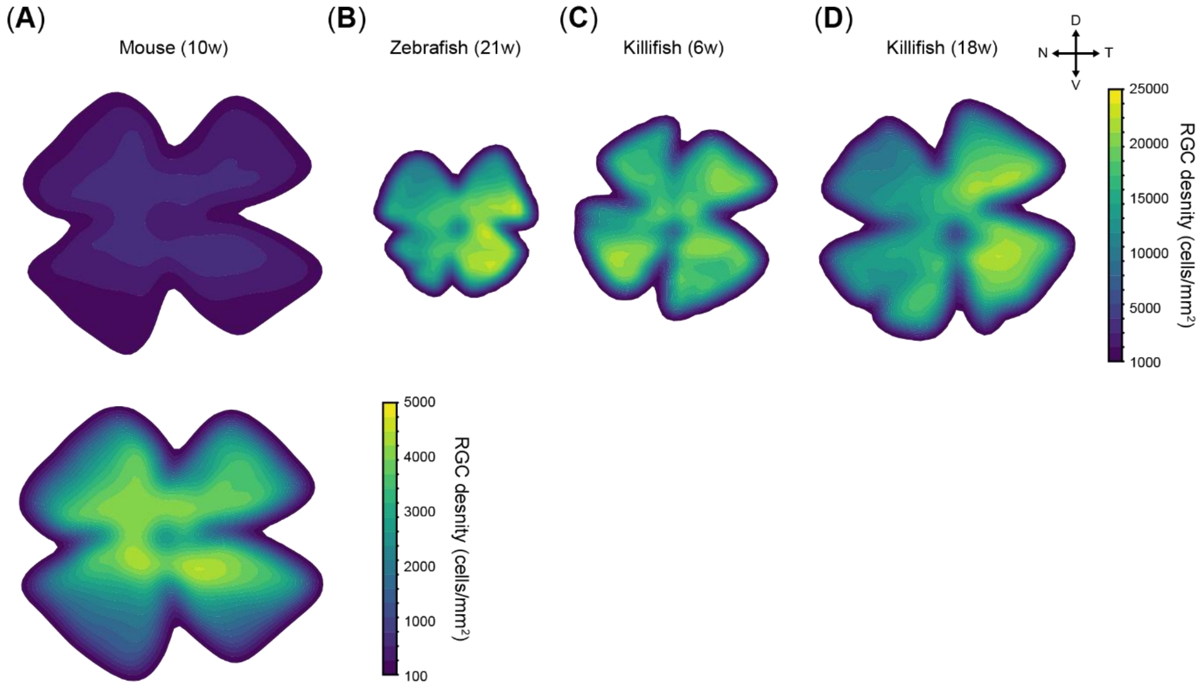

Supplementary figure 2: Scaled isodensity map comparison of non-injured mouse and fish

(**A-D**) Scaled isodensity maps of young adult mouse (10-week-old, **A**), young adult zebrafish (21-week-old, **B**), young adult (6-week-old, **C**) and old (18-week-old, **D**) killifish retinal whole-mounts, showing a considerably higher density of retinal ganglion cells (RGCs) in fish species. (D: dorsal, V: ventral, N: nasal, T: temporal)

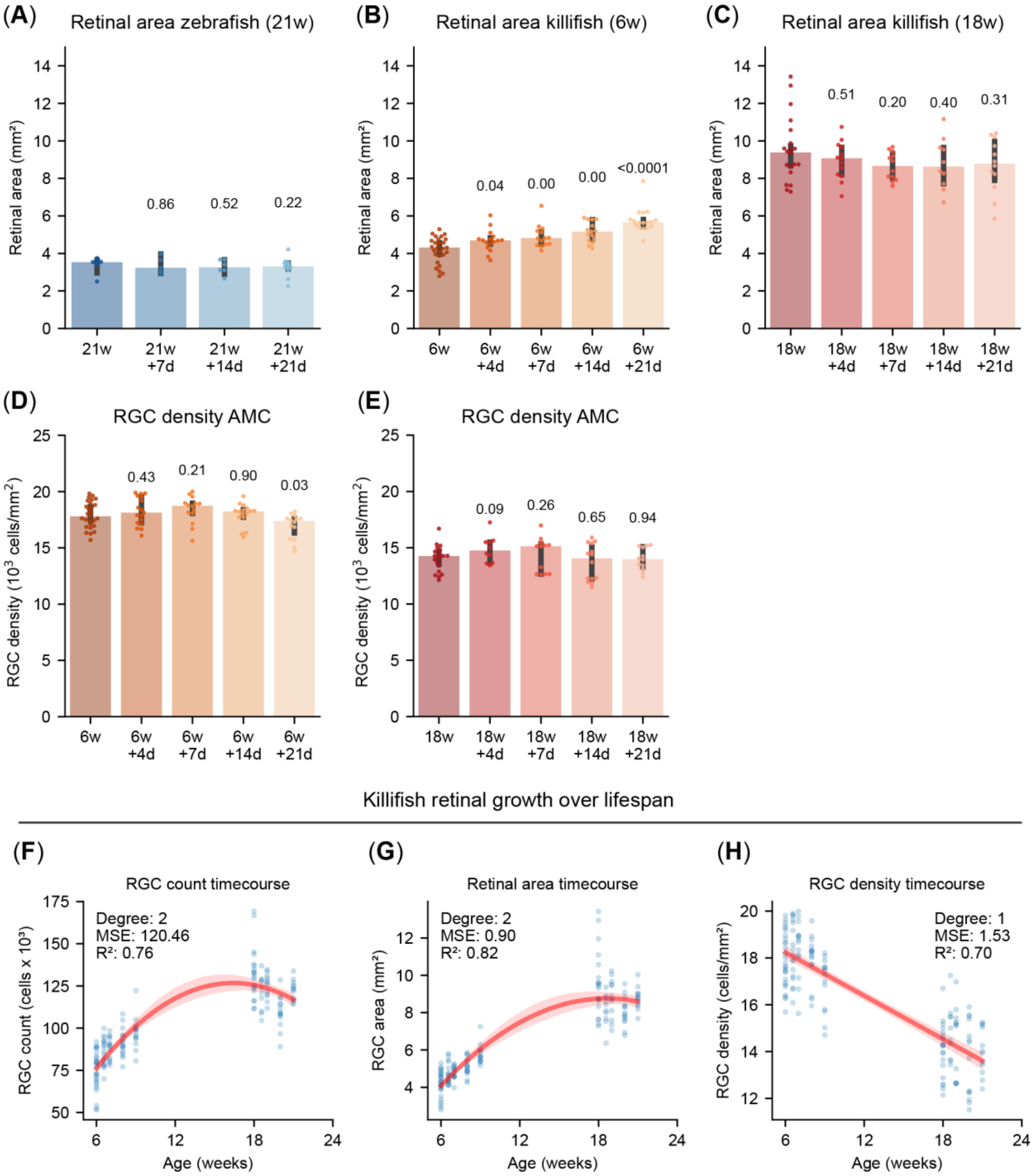

Supplementary figure 3: Retinal growth in non-injured zebrafish and killifish

(**A**) Quantification of retinal area at 21 weeks and at 21 weeks plus 7, 14, and 21days, reveals no significant growth during the experimental time window in zebrafish.

(**B**) Quantification of retinal area in young adult killifish at 6 weeks and at 6 weeks plus 4, 7, 14, and 21days, shows a significant increase in retinal area within the time window of the experiment.

(**C**) Quantification of retinal area in old killifish at 18 weeks and at 18 weeks plus 4, 7, 14, and 21days, does not show significant growth during the experimental time window.

(**D**) Evaluation of retinal ganglion cell (RGC) density in uninjured retinas of killifish along the experimental timeline reveals a significantly lower density of RGCs at 6 weeks plus 21 days

compared to 6 weeks, justifying the usage of age-matched control fish (AMC) for quantitative analyses in young-adult animals.

(**E**) Evaluation of RGC density in uninjured (uninj.) retinas of old killifish along the experimental timeline reveals no significant differences in RGC density in the AMC fish.

(**F-H**) Representation of retinal area (**F**), RGC count (**G**)and RGC (**H**) density across all ages and time points examined in killifish. Models were fitted to capture retinal growth over the lifespan, revealing an approximately linear decline in RGC density with age.

Data from 1 (**A**) or 2 (**B-H**) independent experiments, presented as median ± 25-75th CI (A-E). Kruskal-Wallis ANOVA. P-values reported within the figure. Polynomial models were fit to the data and the degree was chosen based on cross-validation. MSE (Mean squared error)
